# Phytochelatins and Cadmium Mitigation: Harnessing Genetic Avenues for Plant Functional Manipulation

**DOI:** 10.3390/ijms26104767

**Published:** 2025-05-16

**Authors:** Deyvid Novaes Marques, Cássio Carlette Thiengo, Ricardo Antunes Azevedo

**Affiliations:** 1 Department of Genetics, Luiz de Queiroz College of Agriculture (ESALQ), University of São Paulo (USP), Piracicaba, São Paulo 13418-900, SP, Brazil; 2 Luiz de Queiroz College of Agriculture (ESALQ), University of São Paulo (USP), Piracicaba, São Paulo 13418-900, SP, Brazil

**Keywords:** abiotic stress, cadmium, genetic engineering, heavy metals, phytochelatin, phytochelatin synthase, plant biotechnology, tolerance, transgenics

## Abstract

Among the highly toxic heavy metals, cadmium (Cd) is highlighted as a persistent environmental pollutant, posing serious threats to plants and broader ecological systems. Phytochelatins (PCs), which are synthesized by phytochelatin synthase (PCS), are peptides that play a central role in Cd mitigation through metal chelation and vacuolar sequestration upon formation of Cd-PC complexes. PC synthesis interacts with other cellular mechanisms to shape detoxification outcomes, broadening the functional scope of PCs beyond classical stress responses. Plant Cd-related processes have has been extensively investigated within this context. This perspective article presents key highlights of the panorama concerning strategies targeting the PC pathway and PC synthesis to manipulate Cd-exposed plants. It discusses multiple advances on the topic related to genetic manipulation, including the use of mutants and transgenics, which also covers gene overexpression, PCS-deficient and PCS-overexpressing plants, and synthetic PC analogs. A complementary bibliometric analysis reveals emerging trends and reinforces the need for interdisciplinary integration and precision in genetic engineering. Future directions include the design of multigene circuits and grafting-based innovations to optimize Cd sequestration and regulate its accumulation in plant tissues, supporting both phytoremediation efforts and food safety in contaminated agricultural environments.

## 1. Cadmium and Phytochelatins as Strategic Targets

Cadmium (Cd) is a highly toxic pollutant for plants and other living organisms. This heavy metal poses serious risks to human health and is also one of the most extensively studied environmental factors in the field of abiotic stress within the plant research context, frequently occurring under various agricultural conditions (see articles we previously reviewed [1,2]). Here, phytochelatins (PCs), referred to as metal-binding compounds, are highlighted. They are cysteine-rich peptides regarded as key molecules in the detoxification of heavy metals in plants. In response to Cd exposure, plants synthesize PCs, which are able to chelate Cd ions in the cytosol, forming stable complexes. These complexes can then be transported into the vacuole. This mechanism helps lower the toxic concentration of Cd in sensitive cellular compartments [3,4,5]. As such, both the synthesis and chelation capacity of PCs are closely linked to how plants modulate their tolerance and response to Cd upon different experimental settings.

Phytochelatin synthase (PCS) in turn is the key enzyme in the biosynthesis of PCs, catalyzing their formation from the precursor glutathione (GSH) through a transpeptidation reaction involving the polymerization of GSH molecules. PCS activity can be triggered by the presence of heavy metal ions, with Cd recognized as a particularly strong inducer. Its enzymatic activity and gene expression are regulated at both transcriptional and post-transcriptional levels in response to metal exposure and other conditions (see information previously reviewed [3,4,5]). Given its crucial role in metal detoxification, PCS holds significant potential for use in genetic manipulation and genetic engineering applications within the context of heavy metal mitigation.

Considering the growing concern over Cd contamination in agricultural systems and its impact on food safety, there is an increasing demand for innovative and relevant strategies to modulate plant tolerance and control the metal accumulation, along with other relevant topics regarding Cd mitigation. PC and their biosynthetic enzyme, as further presented in the following section, play important roles in Cd detoxification, making them strategic targets for both basic and applied research. This article aims to provide information on the current knowledge of PC-related genetic manipulation to explore how these approaches, along with relevant genetic engineering strategies, may contribute to Cd mitigation in plants, particularly through the enzyme involved. By integrating relevant insights, this paper offers a perspective on the potential of PC-centered approaches in plant biotechnology, also highlighting future research directions in this field.

## 2. Advances in Phytochelatin Synthase Research and Plant Genetic Manipulation

The use of genetic manipulation and various approaches, including mutagenesis and genetic engineering, has established PCS as a relevant and promising genetic target in order to investigate plants with differential tolerance and accumulation of Cd within the context of PCS synthesis. Among such experimental approaches, the use of PCS-deficient mutants, transgenic lines with *PCS* overexpression, and studies that combine both strategies have stood out, in addition to silencing-focused techniques (which include genetic manipulation through RNA interference and virus-induced gene silencing). These approaches have been applied across different Cd-exposed plant species. This includes model organisms such as *Arabidopsis thaliana* and tobacco (*Nicotiana tabacum*), as well as crop species like rice (*Oryza sativa*) and approaches that simultaneously employ both mutants and overexpressing lines (such as the complementation of the *cad1-3* mutant with heterologous *PCS* genes) (Table 1).

The study of PCS-deficient mutants, such as the well-characterized *cad1-3* mutant of *Arabidopsis* (described as deficient in PCS1, containing a defective *AtPCS1* gene), has provided early and robust evidence for the importance of PCS in Cd detoxification. The hypersensitivity of these mutants to Cd directly demonstrated the role of PCS in protecting cells against the toxicity of this metal. Comparing the behavior of *cad1-3* mutants to wild-type plants under Cd exposure made it possible to determine the extent of PCs’ contribution to Cd tolerance and accumulation. Although PCS mutants in general are more sensitive to Cd, many still exhibit some level of tolerance at low concentrations, suggesting the existence of additional metal defense mechanisms [6,7,8,10,13,21].

In transgenic plants, while *PCS* overexpression has, in some cases, led to increased Cd tolerance and accumulation, other studies have reported hypersensitivity (Table 1), indicating that the homeostasis of PCs is finely regulated. Furthermore, different studies have speculated and suggested various reasons for such sensitivity [15,40], which may result from PC toxicity at high concentrations, cytosolic accumulation of PC–Cd complexes exceeding vacuolar sequestration capacity, depletion of GSH, or oxidative stress; and alternatively, excess PCs may be toxic or interfere with alternative detoxification pathways [78].

To provide a clearer understanding of PC-mediated detoxification, it is crucial to distinguish between the regulation of *PCS* gene expression, the actual enzymatic activity of the PCS protein, and the subsequent localization and transport of the synthesized PCs. While PCS gene expression is inducible by heavy metals and exhibits differential spatial and temporal patterns, the activity of the PCS enzyme, which catalyzes PC synthesis from the precursor GSH, is directly triggered by metal ions. Following their synthesis in the cytosol, PCs chelate metals, and the resulting complexes must be transported into the vacuole for effective sequestration (see information previously reviewed [3,4,5]). Crucially, simply increasing *PCS* gene expression or enzyme activity does not guarantee effective detoxification if downstream processes, such as GSH availability or the capacity for vacuolar transport, are limiting. In fact, existing studies demonstrate that PCs play a crucial role in the plant response to Cd, influencing not only tolerance but also the accumulation and translocation of the metal. Even so, *PCS* overexpression does not always lead to consistent, desirable, or positive effects, due to aspects such as the complexity of interactions among different enzyme isoforms, metal transport, and the cellular compartmentalization of Cd-PC complexes (Table 1).

Moreover, Gong et al. [13] and Chen et al. [82], using different experimental approaches (transgenic expression and grafting, respectively) and *cad1-3* plants, provided key evidence supporting the property of PCs to be translocated within plants, their long-distance transport, and their role in the movement and potential detoxification of heavy metals such as Cd. These strategies offer valuable insight into the role of PCS in vivo, but caution is also necessary due to potential pleiotropic effects and agronomic impacts. Although increased Cd tolerance and certain integrative aspects (which includes the modulation of Cd accumulation) may occur, they do not always lead to improvements in desirable agronomic traits or meet agricultural expectations, as we previously discussed [1].

## 3. Research Avenues and Approaches in PC-Related Genetic Manipulation

**Figure 1:**
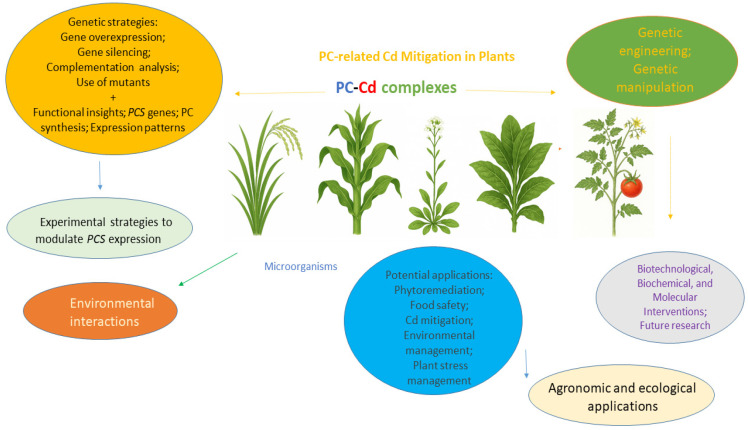
Overview of PC-related Cd mitigation research in plants employing genetic manipulation strategies.

This section outlines key research avenues and emerging perspectives on the use of genetic manipulation strategies to enhance PC-mediated Cd mitigation in plants. An overview of the main approaches investigated to date on this research theme is illustrated in Figure 1.

Other authors have also demonstrated the promising use of synthetic molecules and PC analogs in genetically modified plants as relevant biotechnological tools for research on heavy metal tolerance and remediation [83,84,85]. Postrigan et al. [83] designed and expressed a synthetic pseudophytochelatin gene in tobacco, observing increased resistance to Cd and enhanced accumulation of the metal in the aerial parts. In turn, Zheng et al. [84] investigated a PC-like gene (PCL) encoding a peptide with an α-Glu-Cys linkage, showing improved performance of transgenic tobacco plants under Cd^2+^ stress and greater Cd accumulation, mainly in the roots. The localization of PCL in the cytoplasm and nucleus suggests potential involvement in various physiological processes. Additionally, Vershinina et al. [85] generated transgenic tobacco plants expressing the *pph6his* gene, encoding a PC analog with an α-peptide linkage, which demonstrated stable growth in the presence of Cd and sufficient accumulation in the aerial tissue for Cd mitigation in the given research context. Optimizing the design of these synthetic molecules and analogs with different structures and sizes, exploring regulable and reusable expression systems to enhance phytoremediation efficiency, investigating the transport and complexation mechanisms of these peptides in various plant species and their relevance for Cd mitigation in different agricultural contexts, conducting direct comparative studies between different PC analogs and PCS, evaluating their effectiveness under field conditions and with various types of contamination, as well as analyzing and comparing their short and long-term effects on ecosystems are key perspectives for this research field.

Although PCS1 has historically been the primary focus in this research field, investigations have also explored the role of PCS2 in metal responses under various conditions, including its ability to complement PCS1 function in deficient mutants [17] and the regulation of PCS2 in genetically engineered rice plants (Table 1). Indeed, despite assumptions about PCS1’s primary role in metal tolerance [9], other studies indicate that *Arabidopsis thaliana* PCS2 is constitutively active in vivo [59]. Additionally, the recent study by Li et al. [86] examines the use of both the cad1-3 mutant (linked to the *PCS1* gene) and the pcs2 mutant (associated with *PCS2*) to further support the involvement of these genes in Cd tolerance. Their finding that the overexpression of either *PCS1* or *PCS2* in a *wrky45* mutant background can restore the mutant’s tolerance to Cd stress suggests that both genes participate in the signaling pathway downstream of WRKY45. This evidence was observed in *Arabidopsis thaliana* [86]. Also, in addition to the localization in the cytosol, AtPCS2 was shown to be detected in the nucleus, besides being tightly regulated at the transcriptional level in *Arabidopsis* and potentially playing additional roles beyond PC biosynthesis [87]. Such a regulatory landscape is complex, marked by alternative splicing of genes like *OsPCS2* [71], as well as varied expression patterns dependent on species, exposure time, and metal concentration [Table 1]. Moreover, studies involving the *cad1-3 atpcs2-1* double mutant have shown phenotypes similar to cad1-3 and a lack of detectable PCs, indicating that the low residual PCS activity in cad1-3 is attributable to AtPCS2 [82]. Such findings highlight the ongoing effort to understand the individual and combined contributions of PCS1 and PCS2 isoforms in the complex plant response to metal stress, expanding our knowledge across different plant species as well.

Indeed, PCS2 has been investigated as a functional enzyme capable of mediating PC biosynthesis in response to Cd exposure to some extent, with apparent non-redundancy with AtPCS1 [9]. In *Arabidopsis thaliana*, however, its endogenous expression was notably low due to weak promoter activity and inefficient mRNA translation, limiting its ability to compensate for the Cd hypersensitivity observed in AtPCS1-deficient mutants like *cad1-3* [17]. Despite this, AtPCS2 has shown constitutive PC-synthesizing activity and the capacity to increase Cd accumulation in *cad1-3* mutants under certain conditions [59]. Studies using *PCS*2 genes from other species have yielded contrasting results; overexpression of *MnPCS2* from mulberry in Arabidopsis enhanced Cd tolerance and accumulation [51], whereas AdPCS2 from *Arundo donax* led to increased Cd sensitivity, marked by reduced shoot biomass and chlorosis [52]. In rice, the functional isoform OsPCS2a, distinct from the non-functional OsPCS2b, has been shown to contribute to Cd tolerance and accumulation when expressed in yeast, and its grain-specific silencing via RNAi—alongside OsPCS1—reduces Cd accumulation in rice grains [71]. Moreover, OsPCS2 appears to be the predominant isozyme driving PC synthesis in roots under Cd stress, with its knockdown leading to lower root Cd and PC levels but only marginally affecting whole-plant Cd tolerance [74]. Despite these advances and the contrasting outcomes across species and genetic and experimental contexts, knowledge of PCS2 function within the genetic engineering and manipulation context remains limited to a few plant species and experimental setups, highlighting the need for broader and more systematic investigations as well as including more plant species.

PCS mutants, besides their role in metal detoxification, are involved in processes like nutrient dynamics and pathogen interactions (Table 1). For example, *cad1-3* mutants show altered copper transport, revealing the link between PCs and other metal-binding proteins like metallothioneins (which also comprise other major classes of metal-binding molecules). The triple mutant *mt1a-2 mt2b-1 cad1-3* (showing metallothionein deficiency combined with PC deficiency) exhibited increased sensitivity to Cd and copper, highlighting compensatory or additive roles of different molecules and their contribution to plant metal homeostasis [21]. PC-deficient mutants also have heightened susceptibility to pathogens, linking Cd stress to weakened defense mechanisms, besides exhibiting impaired callose deposition and lignification [31]. These findings suggest that PCS and PC play a broader role in both abiotic and biotic stress responses, besides providing insights into plant survival under combined metal and biotic stress. Future studies could also focus on understanding how PCS mutations and the use of PC-focused transgenes affect plant-pathogen interactions and identify potential compensatory mechanisms in a broader agricultural context.

The study by Cahoon et al. [28] offers a compelling foundation for future biotechnological strategies targeting metal stress tolerance. By using directed evolution, they generated *PCS1* variants in *Arabidopsis thaliana* that, despite reduced catalytic efficiency, conferred enhanced Cd tolerance and accumulation in *Saccharomyces cerevisiae*, *Arabidopsis*, and *Brassica juncea*. These findings suggest that PCS activity can be fine-tuned through targeted mutations, opening new avenues for engineering crops with improved detoxification capacity and resilience to heavy metal stress. Future research may build on this approach to design custom PCS alleles tailored to specific environmental challenges.

Recent investigations have expanded our understanding of PCS and its applications in enhancing Cd tolerance and mitigation in plants. For instance, Chen et al. [33] demonstrated that the heterologous co-overexpression of *SpGSH1* and *SpPCS1* from *Spirodela polyrhiza* enhances both Cd tolerance and accumulation more effectively than individual gene overexpression. In parallel, Gui et al. [78] engineered rice plants with reduced levels of arsenic and Cd in grains through the co-overexpression of *OsPCS1*, *OsABCC1*, and *OsHMA3*, revealing a synergistic reduction in metal accumulation without compromising plant growth. Additionally, Li et al. [86] identified the transcription factor *AtWRKY45* as a positive regulator of Cd tolerance in *Arabidopsis*, acting via upregulation of *PCS1* and *PCS2* expression. Recent studies have also begun to elucidate how PC synthesis is modulated by hormonal cues and integrated with broader physiological responses to Cd stress. Xing et al. [81] for example provided compelling evidence that melatonin enhances Cd tolerance in tomato not only by mitigating oxidative damage but also by directly regulating PC biosynthesis. Using gene silencing of *PCS* and *COMT* (caffeic acid O-methyltransferase), the authors demonstrated that endogenous melatonin levels and PC content are both upregulated under Cd stress, and that melatonin-deficient plants are more susceptible to metal toxicity. These findings highlight a hormonal regulatory layer that influences the antioxidant system, redox homeostasis, nutrient absorption, and ultimately PC-mediated detoxification.

Notably, while *OsPCS1* overexpression alone has been associated with Cd hypersensitivity in some experiments based on rice research [78], combinatorial strategies involving genes encoding metal transporters have shown promise in mitigating this sensitivity and limiting Cd accumulation in edible tissues. This reinforces the notion that multigenic engineering strategies, particularly those that integrate PCS overexpression with genes involved in vacuolar sequestration and long-distance transport, are crucial for balancing detoxification efficiency with agronomic performance.

In this context, mutants such as *cad1-3* (defective in *PCS1*) and *nramp3nramp4* (defective in vacuolar metal remobilization) have provided significant mechanistic insights. As shown by Molins et al. [24], both mutants display enhanced Cd sensitivity, yet their physiological responses diverge under stress. For instance, *nramp3nramp4* exhibits severe damage to the photosynthetic apparatus under Cd exposure, a phenotype not equally observed in *cad1-3*. These findings suggest that vacuolar metal stores play a critical role in safeguarding plastid function during Cd and oxidative stress and highlight the necessity of exploring compensatory pathways when PCS function is impaired.

Taken together, these results underscore the importance of moving beyond single-gene approaches. Future research should prioritize integrative strategies that combine PCS manipulation with regulators of redox homeostasis, vacuolar sequestration, and transcriptional control. The development of synthetic circuits or inducible systems might further fine-tune PC-related responses to Cd stress, minimizing unintended physiological trade-offs and optimizing plant resilience in contaminated environments. Also, such insights can pave the way for advanced bioengineering strategies that combine PC modulation with fine-tuned control over metal transport and distribution, maximizing both tolerance and food safety outcomes in Cd-contaminated environments. Importantly, the study underscores the interconnection between PC biosynthesis and multiple transport-related processes, suggesting that future efforts should explore the cross-talk between PCs and different transport systems, including vacuolar sequestration, long-distance translocation, and efflux mechanisms.

**Figure 2:**
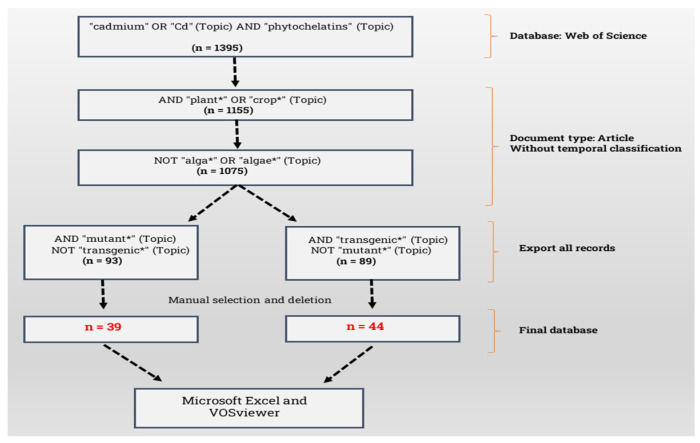
Flowchart for searching and filtering articles on ‘phytochelatins’ and ‘cadmium’ in plants, focusing on studies with ‘mutants’ and ‘transgenics’. * Indicates inclusion and selection criteria used during filtering process.

**Figure 3:**
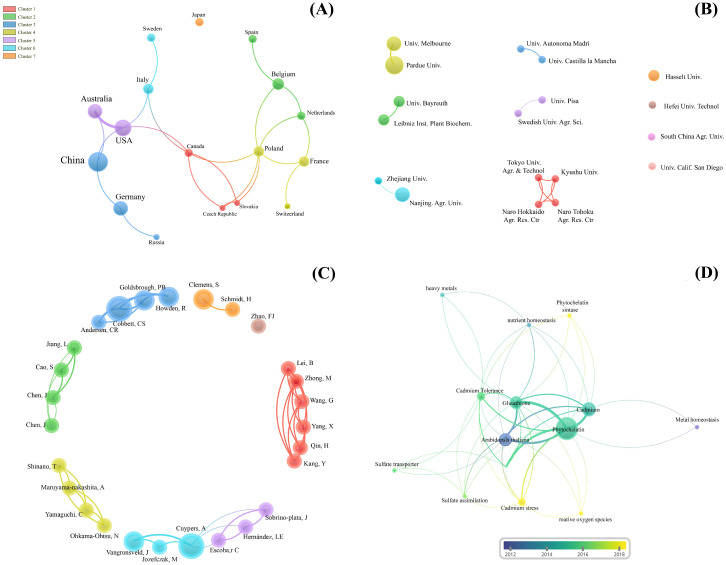
Bibliometric analysis of global participation and research collaboration networks by country (**A**), institutions (**B**), authors (**C**) and keyword co-occurrence network (**D**) in articles on ‘phytochelatins’ and ‘cadmium’ in plants, focusing on studies with ‘mutants’. The size of the circles represents the volume of publications or the frequency of keywords, while the thickness of the lines indicates the strength of the connections or collaborations between the elements analyzed.

**Figure 4:**
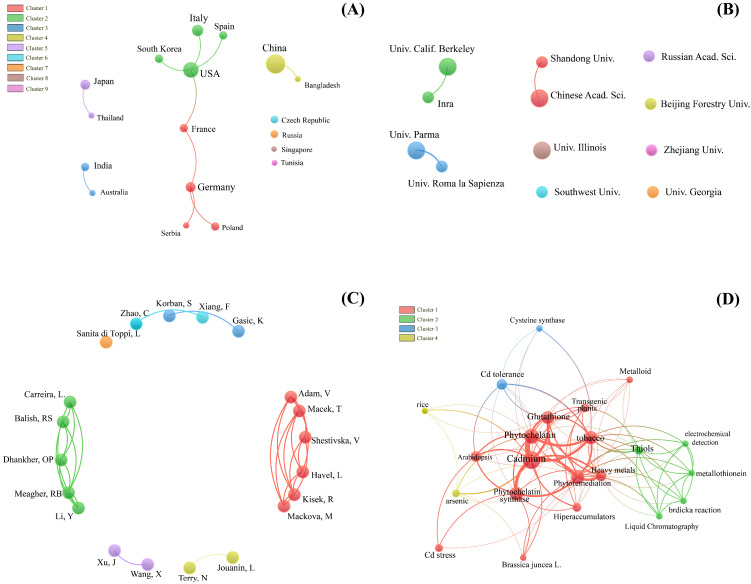
Bibliometric analysis of global participation and research collaboration networks by country (**A**), institutions (**B**), authors (**C**), and keyword co-occurrence network (**D**) in articles on ‘phytochelatins’ and ‘cadmium’ in plants, focusing on studies with ‘transgenics’. The size of the circles represents the volume of publications or the frequency of keywords, while the thickness of the lines indicates the strength of the connections or collaborations between the elements analyzed.

The bibliometric analysis we conducted using the Web of Science Core Collection provided a comprehensive overview of strategies involving PC synthesis in genetically modified plants under Cd stress. Supplementary Material S1 (which also includes Tables S1–S10) provides the full set of information related to this aspect. Using the VOSviewer software (version 1.6.15), we analyzed two subsets of publications: those involving mutants and those focusing on transgenic approaches. The search and filtering process is outlined in the flowchart in Figure 2, while Figure 3 and Figure 4 detail the collaboration networks among countries, institutions, and authors, as well as keyword co-occurrence patterns within each approach.

Our analysis revealed that research in this field extends beyond the classical phytochelatin synthase (PCS) pathway, with recurring terms such as “Glutathione”, “Cadmium”, and “Phytochelatin” pointing to broader strategies for responding to cadmium and other heavy metal stresses. There is a clear opportunity to deepen the integration of the PC pathway with antioxidant mechanisms (such as SOD, CAT, and POX) and with genes involved in Cd-related transport and sequestration (such as ZIP and ABC) (Supplementary Material S1), suggesting promising directions for developing plants with enhanced tolerance and accumulation capacities.

The collaboration networks highlight productive and influential clusters, whose thematic analysis may uncover innovative approaches—such as the modulation of Cd bioavailability in soil or interactions with associated microbiota. These insights increase and contribute to our understanding of effective strategies in genetically modified plants and encourage interdisciplinary practices in the field. Another key point is the frequent use of model organisms like *Arabidopsis thaliana* and *Nicotiana tabacum*, highlighting the need to expand research efforts to agriculturally relevant crops or species with high phytoremediation potential, which holds substantial practical value.

Importantly, we also suggest that future bibliometric analyses take into account not only the content but also the tone and context of the publications, aiming to identify whether a balanced view is presented between the reported benefits and the potential limitations or risks of genetically manipulating the PC pathway. This critical perspective is essential for guiding more mindful, integrated, and sustainable research in plant bioengineering, particularly in the face of heavy metal contamination and its relationship with PC synthesis.

## 4. Concluding Remarks and Additional Future Directions

With regard to future research and ongoing investigations involving PC synthesis, mutant and transgenic plants, and Cd exposure, the integration of diverse approaches is crucial for achieving a more comprehensive and practically relevant understanding. For instance, optimizing transgenic plants through the heterologous expression of *PCS* genes from various species—while also considering factors such as subcellular localization and metabolic balance—represents a promising avenue. Transferring these genetically modified plants into agricultural systems, with an emphasis on food safety, is a critical next step. Furthermore, future studies might prioritize a fuller picture and knowledge regarding tissue-specific analyses and the use of targeted promoters to enhance the precision of transgenic and genetic engineering approaches focused on PC and PCS.

The combination of tools such as transgenic plants with omics approaches, alongside advances in gene editing technologies like CRISPR/Cas9, opens up exciting possibilities for precise modifications of PCS-related pathways. In our research group, we have also been exploring grafting as a strategy to modulate Cd tolerance. Grafting emerges as a powerful methodological innovation to dissect the specific contributions of root and shoot systems in response to Cd exposure, enabling investigations into inter-organ signaling and the modulation of metal accumulation in targeted tissues.

We have been working on the integration of grafting [1,2] with omics approaches [1,88,89] in plants, which potentially can help uncover the molecular mechanisms underlying Cd tolerance and transport related to PC synthesis. These different perspectives align with the future directions we have discussed, highlighting the importance of refining transgenic approaches, manipulating transcription factors, and applying grafting techniques to drive more robust advancements in engineering Cd-tolerant plants.

There is also a clear need for more comprehensive studies that simultaneously assess Cd accumulation, growth and productivity parameters, and the underlying mechanisms of tolerance and response. Broadening the focus to include other cellular components involved in metal homeostasis—such as ABC (ATP-binding cassette transporters) transporters and transcription factors—is equally essential.

Combining genetic manipulation of the PC pathway with agronomic strategies to modulate systemic responses holds strong potential to drive significant progress in both phytoremediation efforts and the production of safe food.

From the integrated analysis of available studies, a clear direction emerges: the development of multi-target precision engineering approaches. These would combine targeted gene editing of specific *PCS* isoforms with the simultaneous modulation of key genes involved in the transport and compartmentalization of Cd-PC complexes. The studies summarized in the Table also reveal inconsistent patterns of Cd accumulation and tolerance depending on genetic, tissue-specific, and metabolic contexts, highlighting that simple *PCS* overexpression is often insufficient to ensure consistent responses.

The given information reinforces the need for modular editing systems capable of fine-tuning not only *PCS* levels but also genes such as *ABCC1/2/3*, *HMA2/4*, and redox-related elements like *GSH1* and *YCF1*, depending on the plant species, target organ, and intended goal (phytoremediation vs. food safety). Technologies such as multiplex CRISPR/Cas9 and expression vectors with tissue-specific and/or stress-inducible promoters offer the level of precision required for such strategies.

Moreover, applying these approaches in model platforms—such as transgenic lines grafted with cultivated plant tissues—could serve as a valuable translational validation system before deploying them in economically relevant food crops. These proposals point toward a future where Cd tolerance can be modulated in a dynamic, efficient, and highly specific manner, tailored to the ecological and agricultural demands of each unique scenario.

## Figures and Tables

**Table 1:** Examples of functional genetic manipulations for Cd mitigation in plants, focusing on PCS-related insights and modulation of PC synthesis.

Overall Experimental Genetic Approach	Genes; Locus; Allelic Series	Examples of Main Findings Related to PCs in Cd-Exposed Plants	Citation
Isolation and characterization of Cd-sensitive mutants	*CAD1* locus (*cad1-1*, *cad1-2*)	Mutants show reduced Cd sequestration, but BSO (PC biosynthesis inhibitor) further increases sensitivity non-additively, suggesting PCs are still produced and *cad1* affects a related detoxification aspect.	[6]
Isolation and characterization of Cd-sensitive *cad1* mutants	*CAD1* locus (*cad1-1*, *cad1-2*, *cad1-3*, *cad1-4*, *cad1-5*)	Mutants show deficient PC accumulation and PCS activity correlating with increased Cd sensitivity; *cad1-3* (most sensitive) shows no detectable PCs; BSO increases wild-type Cd sensitivity but has little effect on *cad1-3*, suggesting a primary role for PCs in Cd detoxification.	[7]
Positional cloning and characterization of *cad1* mutants; functional complementation; heterologous expression in *E. coli*	*CAD1* gene (MRH10.11)	*cad1* mutants are Cd-sensitive and PC-deficient with lack of PC synthase activity; *CAD1* encodes a GSH-dependent, heavy metal-activated PC synthase; *CAD1* mRNA expression is not induced by Cd.	[8]
Functional characterization and expression analysis of a second *PCS* gene in *Arabidopsis thaliana* through heterologous expression in yeast and comparison with *cad1* mutants	*AtPCS2*, *AtPCS1* (*CAD1*)	*AtPCS2* encodes a functional PC synthase in yeast, but *cad1* mutants (lacking functional *AtPCS1*) show no detectable PCs upon Cd exposure in *Arabidopsis*, indicating non-redundancy and that *AtPCS1* is likely the primary Cd-inducible PC synthase in *Arabidopsis*.	[9]
Comparison of Cd uptake and accumulation in PC-deficient *cad1-3* mutant and wild type *Arabidopsis* with and without prior Cd exposure.	*cad1-3* (deficient in PC synthase activity)	The PC-deficient mutant accumulated less Cd than the wild type at higher growth Cd concentrations, with lower Cd translocation to the shoot, suggesting PCs enhance Cd uptake and translocation.	[10]
Characterization of AtPCS1 promoter activity using GUS reporter gene in transgenic *Arabidopsis*; analysis of AtPCS1 protein accumulation using FLAG-tagged constructs; investigation of Cd’s effect on AtPCS1 protein levels; comparison of expression using genomic DNA vs. cDNA of *AtPCS1*.	*AtPCS1*	*AtPCS1* promoter shows differential spatial and temporal activity, including strong expression in leaf trichomes; AtPCS1 protein levels correlate with promoter activity; AtPCS1 protein expression is not post-transcriptionally regulated by Cd; introns enhance *AtPCS1* mRNA and protein accumulation.	[11]
Overexpression of wheat *PCS* gene (*TaPCS1*) in *Nicotiana glauca*	*TaPCS1*	Transgenic *N. glauca* seedlings showed increased tolerance to Cd (longer roots and greener leaves) compared to wild-type, suggesting enhanced PC activity due to *TaPCS1* overexpression.	[12]
Expression of wheat *TaPCS1* cDNA in Arabidopsis *cad1-3* mutant (root-specific and ectopic promoters); analysis of Cd transport and PC levels	Wheat *TaPCS1*, Arabidopsis *AtPCS1* (*cad1-3* locus)	Root-specific *TaPCS1* expression leads to PC transport from roots to shoots, enhanced root-to-shoot Cd transport, and reduced Cd accumulation in roots, all of which are PC-dependent.	[13]
Generation and characterization of transgenic *Arabidopsis* overexpressing *AtPCS1* under the CaMV 35S promoter; analysis of Cd tolerance, accumulation, and PC production	*AtPCS1*	High levels of *AtPCS1* overexpression led to increased PC production but did not necessarily enhance Cd tolerance or accumulation, and in some cases caused increased Cd sensitivity; moderately overexpressing lines showed increased tolerance and accumulation.	[14]
Overexpression of *AtPCS1* in transgenic *Arabidopsis* under the control of the *AtPCS1* promoter	*AtPCS1*	Overexpression led to increased *AtPCS1* mRNA and PC production under Cd stress but paradoxically resulted in hypersensitivity to Cd and Zn (not Cu), possibly due to toxicity of PCs at supraoptimal levels compared to GSH.	[15]
Overexpression of *AtPCS1* in *Arabidopsis thaliana*	*AtPCS1*	Overexpression led to increased PC levels upon Cd exposure but surprisingly resulted in Cd hypersensitivity compared to wild-type plants.	[16]
Expression analysis and complementation studies of AtPCS2 in the AtPCS1-deficient *cad1-3* mutant using various promoters	*AtPCS2*, *AtPCS1* (*CAD1*), *cad1-3*	*AtPCS2* expression is weak compared to *AtPCS1* and cannot fully complement the *cad1-3* mutant’s Cd sensitivity even when overexpressed, suggesting low expression levels limit its role in PC production in this context.	[17]
Transcript level analysis of AtMRPs under Cd treatment using wild-type and mutant plants	*AtMRP3*, *cad2* (*GSH deficient*), *cad1-3* (*PC deficient*)	AtMRP3-related induction by Cd is likely independent of GSH and PC biosynthesis/levels.	[18]
Analysis of Cd sensitivity in PC-deficient Arabidopsis mutants	*cad1-3* (*PCS deficient*)	Deficiency in PC production leads to increased sensitivity to Cd.	[19]
Analysis of Cd sensitivity and root-to-shoot translocation using hma2, hma4, and cad1 single and multiple mutants	*HMA2*, *HMA4*, *CAD1*; *hma2-4*, *hma4-2*, *cad1-3*	PC deficiency resulted in increased shoot Cd and percentage translocation, but had a minor effect compared to hma2 and hma4 loss.	[20]
Analysis of Cd tolerance in metallothionein and PC-deficient Arabidopsis mutants	*cad1-3*, *mt1a-2*, *mt2b-1*, *mt1a-2, mt2b-1, cad1-3*	Combined deficiency of PCs and MTs (MT1a, MT2b) leads to increased sensitivity to Cd.	[21]
Analysis of Cd sensitivity in Arabidopsis PC-deficient mutants	*AtPCS1*; *cad1-3*, *cad1-6*	PC deficiency leads to pronounced hypersensitivity to Cd.	[22]
Characterization of knockout mutants (single and double) and overexpression lines in Arabidopsis; heterologous expression in yeast.	*AtABCC1* (*At1g30400*); *AtABCC2* (*At2g34660*); *atabcc1*, *atabcc2*, *atabcc1 atabcc2* (*knockouts*); *AtABCC1* (*overexpression*)	AtABCC1 and AtABCC2 are important for vacuolar sequestration of PC–Cd(II), conferring tolerance; atabcc1 atabcc2 shows reduced vacuolar Cd.	[23]
Characterization of a loss-of-function mutant	*PCS1* (*CAD1*); *cad1-3*	Loss of PCS1 function leads to Cd hypersensitivity due to the inability to synthesize PCs under Cd stress.	[24]
Characterization of a T-DNA tagged knockout mutant in Arabidopsis; in silico 3D structure prediction and in vivo metal accumulation analysis.	*PCS1* (*At5g44070*); *SAIL_650_C12* (*homozygous T-DNA insertion mutant*)	The *pcs1* mutant (SAIL_650_C12) shows higher Cd accumulation in shoots and increased Cd sensitivity due to the inability to synthesize PCs.	[25]
Characterization of knockout mutants	*PCS* (*non-functional in cad1-3*); *cad2-1*, *rax1-1*, *cad1-3*	*cad1-3* mutant deficient in PCS shows no PC accumulation under Cd exposure, confirming its role in PC synthesis; Cd is a stronger inducer of PCs than Hg.	[26]
Characterization of knockout mutant (abcc3) and overexpression lines (AtABCC3ox) in Arabidopsis; complementation assay in atabcc1 atabcc2 mutant; pharmacological inhibition of PC synthesis (BSO); analysis of Cd localization using fluorescent dyes.	*AtABCC3* (*AtMRP3*); *abcc3* (*knockout*); *AtABCC3ox* (*overexpression*); *cad1-3* (*PCS deficient*); *atabcc1 atabcc2* (*double knockout*)	AtABCC3 is involved in vacuolar transport of PC–Cd complexes, contributing to Cd tolerance; abcc3 mutants are Cd-sensitive with reduced vacuolar Cd; *AtABCC3ox* lines show increased Cd tolerance and vacuolar Cd; AtABCC3 function in Cd tolerance is PC-dependent and can complement atabcc1 atabcc2 in the presence of PCs.	[27]
Directed evolution of *Arabidopsis thaliana* PCS (AtPCS1); heterologous expression of wild-type and mutant AtPCS1 in yeast (Saccharomyces cerevisiae); ectopic expression of wild-type and mutant AtPCS1 in Arabidopsis thaliana and Brassica juncea.	*AtPCS1* (*GenBankTMAF085230*); *various mutant variants* (e.g., *Y186C*)	Specific *AtPCS1* mutants with diminished catalytic activity conferred improved Cd tolerance and increased PC accumulation in yeast, *Arabidopsis*, and *B. juncea*. This was linked to the maintenance of glutathione and γ-glutamylcysteine precursor levels and redox homeostasis during Cd exposure, unlike the overexpression of wild-type *AtPCS1*, which could deplete these metabolites.	[28]
Characterization of Arabidopsis AtPCS1 knockout mutants (*cad1-3* and *cad1-6*) grown on Cd-contaminated soil; heterologous expression of truncated AtPCS1 in yeast.	*AtPCS1*; *cad1-3* (*null mutant*), *cad1-6* (*T-DNA insertion*)	*cad1-3* shows Cd hypersensitivity and no detectable PC accumulation; *cad1-6* is more Cd tolerant with trace PCs in leaves; both mutants have reduced leaf Cd; truncated AtPCS1 can retain Cd-activated PC synthesis.	[29]
Characterization of a PC-deficient knockout mutant (*cad1-3*) in Arabidopsis; comparison with wild-type under Cd exposure (with/without supplemental Cu).	*PCS1* (*CAD1*); *cad1-3*	*cad1-3* mutant lacking PCs is more sensitive to Cd; Cd induces a Cu deficiency response dependent on PCs (reduced in *cad1-3*); PCs likely complex both Cd and Cu; *cad1-3* shows altered metal accumulation and reduced Cd translocation.	[30]
Comparison of Arabidopsis thaliana wild-type and the *cad1-3* knockout mutant (deficient in AtPCS1) under control and Cd exposure conditions.	*PCS1* (*AtPCS1*); *cad1-3*	*cad1-3* mutant shows defective callose deposition after Cd exposure and increased Cd sensitivity, suggesting AtPCS1 contributes to Cd tolerance not only through PC synthesis but also via callose deposition.	[31]
Forward genetics to isolate a Cd-hypersensitive mutant in PC-deficient (cad1-3) background; characterization of the identified PP2A-4C mutant and its interaction with ethylene production under Cd stress.	*PCS1* (*CAD1*); *cad1-3* (*PC-deficient mutant*); *PP2A-4C*; *cdsr1* (*allele isolated in cad1-3 background*); *pp2a-4c-1* (*T-DNA insertion mutant for PP2A-4C*).	The study used a PC-deficient mutant (*cad1-3*) to explore Cd toxicity responses beyond the PC pathway. The identified *PP2A-4C* mutation exacerbated Cd sensitivity in the *cad1-3* background, indicating a PC-independent mechanism of Cd tolerance modulated by PP2A via ethylene production.	[32]
Forward genetic screen for Cd-hypersensitive mutants in PC-deficient (cad1-3) background; identification and characterization of cdsr2 mutant with a mutation in IAR4; analysis of Cd accumulation and thiol compound biosynthesis (including PCs).	*PCS1* (*CAD1*); *cad1-3* (*PC-deficient mutant*); *IAR4*; *cdsr2* (*allele isolated in cad1-3 background*); *cdsr2 cad1-3* (*double mutant*); *iar4-2*, *iar4-8* (*T-DNA insertion mutants for IAR4*).	The study used the PC-deficient *cad1-3* mutant to isolate further Cd-sensitive mutants, revealing that IAR4 mutation exacerbates Cd sensitivity independently of PC synthesis. In the Col-0 background, IAR4 mutation led to increased Cys, GSH, PC2, and PC3 under Cd stress, suggesting a heightened stress response. As expected, PCs were undetectable in *cad1-3* and *cdsr2 cad1-3* under Cd stress due to a defective PCS1 gene.	[33]
Expression analysis (RT-PCR, promoter-LUC assays), protein analysis (Western blot), characterization of sbp1 null mutant and SBP1 overexpression lines in wild-type and PC-deficient (cad) Arabidopsis, and in ycf1 yeast mutant; in vitro Cd binding assays of SBP1.	*SBP1*, *SBP2*, *SBP3*; *sbp1* (*null mutant*); *SBP1* (*overexpression lines*); *PCS1* (*CAD1*) (*cad mutants lacking PCs*); *YCF1* (*Saccharomyces cerevisiae mutant*).	*SBP1* expression is induced by Cd. The sbp1 mutant showed no clear phenotype, possibly due to SBP2 upregulation. Overexpression of SBP1 enhanced Cd accumulation in roots but reduced Cd sensitivity in wild-type and, notably, in cad mutants (lacking PCs), suggesting a PC-independent Cd detoxification role potentially through direct Cd binding.	[34]
Overexpression of *AtPCS1* (cytosol and chloroplast targeting)	*AtPCS1* from *Arabidopsis thaliana*, targeted to cytosol and chloroplast.	Increased PC production upon Cd exposure; cytosolic overexpression led to Cd hypersensitivity; plastidial overexpression showed no significant effect on Cd tolerance.	[35]
Overexpression of *AtPCS1* in *Nicotiana tabacum*	*AtPCS1* from *Arabidopsis thaliana* expressed in tobacco.	Increased PC production and Cd tolerance/accumulation in roots and shoots (GSH-dependent), but no enhanced Cd translocation to the shoot.	[36]
Overexpression of *E. coli cysE*, *E. coli gshA*, and *S. pombe PCS*	Genes involved in cysteine and glutathione biosynthesis from *E. coli* and PC synthase from *Schizosaccharomyces pombe*.	Increased non-protein thiols; higher Cd concentration in roots, no significant increase in shoots after Cd exposure.	[37]
Overexpression of *CdPCS1* in tobacco	*CdPCS1* from *Cynodon dactylon* expressed in tobacco.	Increased PC levels and enhanced Cd accumulation and tolerance in transgenic plants.	[38]
Overexpression of *AtPCS1* in *Arabidopsis thaliana*	*AtPCS1* from *Arabidopsis thaliana* (pcs9 line).	Overexpression did not increase the maximum capacity for non-protein thiol (NPT) production upon Cd exposure; transgenic line showed Cd hypersensitivity.	[39]
Overexpression of *AtPCS1* in *N. tabacum*	*AtPCS1* from *Arabidopsis thaliana* expressed in tobacco.	Increased PCS activity, moderate PC increase, strong γ-EC accumulation, GSH depletion in leaves led to Cd hypersensitivity.	[40]
Simultaneous overexpression of *AsPCS1* and *GSH1* in *A. thaliana*	*AsPCS1* (Arsenate reductase-coupled PCS 1) and *GSH1* (Glutathione synthetase) from an unspecified source, co-expressed in *Arabidopsis*.	Dual-gene transgenic lines showed higher Cd accumulation but not the highest PC content under Cd exposure compared to single-gene overexpressing lines and wild-type.	[41]
Overexpression of *AtPCS1* in *Nicotiana tabacum* (lines PaII12)	*AtPCS1* from *Arabidopsis thaliana* expressed in specific transgenic lines of tobacco.	Decreased cytosolic and vacuolar PC levels in Cd-exposed plants led to Cd hypersensitivity despite no significant change in vacuolar Cd accumulation compared to wild-type.	[42]
Overexpression of *AtPCS1* in *Arabidopsis*	*AtPCS1* from *Arabidopsis thaliana* expressed in *Arabidopsis*.	Increased PC content in *AtPCSox* lines upon Cd exposure; lower Cd tolerance at low Cd but higher tolerance at high Cd compared to wild type; exogenous GSH did not increase Cd tolerance in *AtPCSox* lines.	[43]
Overexpression of *TcPCS1* in *N. tabacum*	*TcPCS1* from *Thlaspi caerulescens* expressed in tobacco.	Enhanced PC production and increased Cd accumulation in roots and shoots; longer root length under Cd stress.	[44]
Simultaneous overexpression of *AsPCS1* and *YCF1* in *A. thaliana*	*AsPCS1* (Arsenate reductase-coupled PCS 1) and *YCF1* (vacuolar membrane protein involved in heavy metal tolerance) from unspecified sources, co-expressed in *Arabidopsis*.	Dual-gene lines showed higher PC content after Cd exposure and higher Cd accumulation than single-gene lines and wild type, indicating increased tolerance.	[45]
Heterologous overexpression of *NnPCS1* in *A. thaliana*	*NnPCS1* from *Nelumbo nucifera* expressed in *Arabidopsis thaliana*.	Increased PC content and enhanced Cd accumulation in transgenic *Arabidopsis* plants.	[46]
Heterologous overexpression of *CdPCS1* in *Arabidopsis thaliana*	*CdPCS1* from *Cynodon dactylon* expressed in *Arabidopsis thaliana*.	Enhanced accumulation of heavy metal(loid)s including Cd in aerial parts without significant growth difference compared to wild type, suggesting potential for phytoremediation.	[47]
Overexpression of *PtPCS* in *N. tabacum*	*PtPCS* from an unspecified plant source expressed in tobacco.	Transgenic plants showed enhanced Cd tolerance and higher Cd accumulation in roots and leaves upon Cd exposure compared to wild type, but a lower transfer coefficient.	[48]
Overexpression of *NtPCS1* in *Nicotiana tabacum* (sense lines)	*NtPCS1* from *Nicotiana tabacum* overexpressed in tobacco.	Enhanced Cd tolerance but no significant change in Cd accumulation was observed compared to control plants.	[49]
Expression of *NtPCS1* in *Nicotiana tabacum* (antisense lines)	*NtPCS1* from *Nicotiana tabacum* underexpressed in tobacco.	Showed growth retardation in the early stage suggesting a role in plant development; PC levels under Cd exposure were not explicitly detailed.	[49]
Overexpression of *AtPCS1* in *Nicotiana tabacum* (*rolB-AtPCS1*)	*AtPCS1* from *Arabidopsis thaliana* expressed in tobacco lines with the *rolB* gene.	Transgenic plants showed higher PC levels and increased Cd accumulation in roots when exposed to Cd, but inhibited Cd extrusion from leaves.	[50]
Overexpression of *MnPCS1* and *MnPCS2* in *Arabidopsis thaliana*	*MnPCS1* and *MnPCS2* from *Mimulus guttatus* expressed in *Arabidopsis thaliana*.	Transgenic *Arabidopsis* lines showed higher Cd accumulation in shoots and roots compared to wild type when grown in Cd-containing medium, suggesting enhanced PC activity led to increased metal uptake and sequestration.	[51]
Overexpression of *AdPCS2* or *AdPCS3* in *Arabidopsis thaliana*	*AdPCS2* and *AdPCS3* from *Arundo donax* expressed in *Arabidopsis thaliana*.	Transgenic *Arabidopsis* lines exhibited significant growth reduction and chlorosis when treated with Cd, suggesting that the overexpression of these *A. donax* PCS genes can lead to enhanced Cd sensitivity in *A. thaliana*.	[52]
Overexpression of truncated *IpPCS1* in *Arabidopsis*	Truncated *IpPCS1* from *Ipomoea pes-caprae* expressed in *Arabidopsis*.	Transgenic seedlings showed slightly improved Cd tolerance, but adult plants displayed no obvious effects on growth or Cd accumulation compared to the wild type under Cd stress.	[53]
Overexpression of *BnPCS1* from *Boehmeria nivea* in *Arabidopsis thaliana*	*BnPCS1* from ramie expressed in *Arabidopsis thaliana*.	Transgenic *Arabidopsis* showed enhanced Cd tolerance, higher Cd accumulation in shoots, and an increased translocation factor of Cd from roots to shoots compared to wild type under Cd stress, suggesting improved phytoremediation potential.	[54]
Transformation of *cad1-3* mutant and wild type *Arabidopsis* with leaf-specifically expressed *AtPCS1*	*AtPCS1; cad1-3*	Leaf-specific AtPCS1 restored leaf PCS activity and PC production in *cad1-3* without increasing total leaf PC levels compared to wild type, increased Cd tolerance but did not limit Cd accumulation to leaves	[55]
Analysis of cad1-3 mutant and transgenic Arabidopsis ectopically expressing wheat *TaPCS1*	*AtPCS1* *;* *cad1-3* *;* *TaPCS1*	Transgenic plants showed higher Cd-PC2 levels in roots and shoots than wild type; more Cd-PC2 found in shoots than roots in both, indicating PC contribution to shoot Cd accumulation	[56]
Complementation of *Arabidopsis cad1-3* mutant and overexpression in transgenic tobacco with CdPCS1 from *Ceratophyllum demersum*	*CdPCS1* *;* *AtPCS1* *(* *CAD1* *);* *cad1-3*	Transgenic tobacco showed increased PC content and enhanced Cd accumulation; CdPCS1 complemented *cad1-3* Cd sensitivity.	[57]
Expression of synthetic PC (EC) genes in wild type and PC-deficient *cad1-3* mutant *Arabidopsis*	*EC14* *,* *EC16* *,* *EC20* *;* *AtPCS1* *(* *CAD1* *);* *cad1-3*	Expression of ECs complemented *cad1-3* Cd sensitivity; transgenic plants showed enhanced heavy metal(loid) accumulation (including Cd) compared to controls.	[58]
Analysis of AtPCS2 overexpression in cad1-3 mutant to assess Cd tolerance and PC accumulation	*AtPCS2* overexpression in AtPCS1 (*CAD1*) mutant *cad1-3*	Overexpression led to constitutive PC2 production, partial rescue of *cad1-3* Cd hypersensitivity on soil, and reduced PC3 levels upon Cd exposure compared to AtPCS1 overexpression	[59]
Expression of C. elegans *CePCS* in Arabidopsis *AtPCS1*-deficient *cad1-3* mutant	*AtPCS1* (CAD1); *cad1-3*; *CePCS*	*CePCS* expression in *cad1-3* restored PC synthesis and Cd tolerance	[60]
Overexpression of *Vicia sativa* PCS1 homolog (*VsPCS1*) in wild-type and AtPCS1-deficient *Arabidopsis* (*atpcs1*)	*VsPCS1* *;* *AtPCS1* *;* *atpcs1*	*VsPCS1* overexpression increased PC synthesis and Cd tolerance; complemented *atpcs1* PC deficiency and Cd sensitivity; led to higher-order PCs (PC4).	[61]
Overexpression of three duplicated *BnPCS* genes from *Brassica napus* in *Arabidopsis thaliana cad1–3* mutant.	*BnPCS* (three duplicated genes); *AtPCS1* (*CAD1*); *cad1-3*	Transgenic lines showed higher PC content and enhanced Cd tolerance, accumulation, and translocation compared to *cad1-3* under Cd stress.	[62]
Overexpression of maize *ZmPCS1* in *Arabidopsis* and complementation of *Arabidopsis atpcs1* mutant	*ZmPCS1*; *AtPCS1*; *atpcs1*	Overexpression in *Arabidopsis* enhanced Cd tolerance and accumulation, along with increased GSH and PC contents; *ZmPCS1* rescued the Cd-sensitive phenotype of the *atpcs1* mutant.	[63]
Isolation and functional characterization of SepPCS from *Sedum plumbizincicola* by complementation in yeast and *Arabidopsis cad1-3* mutant	SepPCS (from *S. plumbizincicola*); AtPCS1 (*CAD1*); cad1-3	*SepPCS* expression restored PCs biosynthesis and Cd tolerance in mutants; PCs’ role in *S. plumbizincicola* Cd tolerance might increase with elevated Cd.	[64]
RNAi silencing of PCS in rice seeds.	OsPCS1 (GenBank AF439787); ZMM1 (seed-specific promoter).	*OsPCS1* silencing in seeds reduced Cd accumulation by ~50%.	[65]
Overexpression of Arabidopsis PCS in Indian mustard.	*AtPCS1* cDNA under the 35S promoter.	Moderate *AtPCS1* overexpression increased PCs and Cd tolerance but decreased Cd accumulation.	[66]
Overexpression of Arabidopsis PCS in Indian mustard.	AtPCS1 gDNA under its native promoter.	Higher PC levels under Cd stress and lower Cd accumulation in shoots with enhanced Cd tolerance.	[67]
Overexpression of *Arabidopsis PCS* in rice.	*AtPCS* gene; hpt II marker gene; *Oryza sativa* genotype Azucena.	Enhanced Cd tolerance, increased PC content, reduced GSH depletion, higher root Cd.	[68]
Heteroexpression of wheat PCS in rice.	*TaPCS1* cDNA under the 35S promoter; *Oryza sativa* L., cv. Zhonghua 11.	Enhanced Cd sensitivity, increased shoot Cd, higher PC and NPT in shoots under Cd.	[69]
Heterologous expression of Phragmites australis PCS in tall fescue.	*PaPCS* cDNA under the 35S promoter.	Higher PC levels, enhanced Cd tolerance, and increased Cd accumulation.	[70]
Seed-specific RNAi to silence *OsPCS* genes.	*OsPCS1*; *OsPCS2*; endosperm-specific GluC promoter.	*OsPCS1* and *OsPCS2* silencing in rice grains led to ~51% reduction in Cd content.	[71]
Characterization of a PCS mutant.	*OsPCS1* (Os05g0415200); has2 (three-base deletion in catalytic domain).	*has2* mutant (impaired OsPCS1) showed similar Cd tolerance and grain Cd content to wild type.	[72]
Characterization of OsPCS1 loss-of-function mutants (T-DNA and Tos17 insertion lines) in rice.	OsPCS1 (Os06g0102300/LOC_Os06g01260); T-DNA insertion; Tos17 insertion (NG5045).	*OsPCS1* mutants showed increased Cd sensitivity, decreased Cd in shoots/grains, reduced PCs under Cd.	[73]
RNAi-mediated silencing of *OsPCS2* expression.	*OsPCS2* gene; constitutive maize ubiquitin1 promoter.	*OsPCS2*-silenced plants showed reduced PC levels under Cd stress, but Cd tolerance was little affected.	[74]
Overexpression of two rice *PCS* genes in Arabidopsis thaliana.	*OsPCS5; OsPCS15*; under the CaMV 35S promoter.	Overexpression paradoxically increased sensitivity to Cd, possibly due to excess PC.	[75]
Functional characterization of the first *PCS* gene from a liverwort using yeast and Arabidopsis cad1-3 complementation.	*MpPCS* (Mapoly0046s0028.1); cad1-3 (Arabidopsis AtPCS1 null mutant).	*MpPCS* overexpression in cad1-3 complemented Cd sensitivity and restored PC2/PC3 production.	[76]
Generation and characterization of *M. polymorpha* knockout mutants and overexpressing lines for *PCS*.	*MpPCS* gene; CRISPR/Cas9 knockout (four alleles); overexpression lines (MpPCS-ox-3, MpPCS-ox-7).	*Mppcs* mutants Cd hypersensitive, no PC; overexpressors also Cd hypersensitive, reduced GSH/PCn, higher Cd accumulation.	[77]
Overexpression of a rice *PCS* gene.	OsPCS1 (Os05g0415200) cDNA under the rice OsActin1 promoter.	Increased PC, decreased grain Cd, but caused seedling Cd hypersensitivity by suppressing vacuolar Cd sequestration.	[78]
Heterologous expression of cyanobacterial PCS in *Artemisia annua* hairy roots.	*anaPCS* gene (from Anabaena PCC 7120) under CMV35S promoter.	Transformed hairy roots showed significantly higher PC content and greater Cd accumulation.	[79]
Overexpression of *Tamarix hispida PCS* gene.	*ThPCS1* gene (full length, 1581 bp ORF) from *Tamarix hispida*; pROKII-ThPCS1 vector.	ThPCS1 overexpressors showed less reactive oxygen species and Cd ions under Cd stress.	[80]
VIGS of *PCS* in tomato.	*PCS*	PCS silencing aggravated Cd phytotoxicity; melatonin-induced Cd tolerance partly depends on PCs.	[81]

The following abbreviations are used in the table: AdPCS2 = *Arundo donax* phytochelatin synthase 2; AdPCS3 = *Arundo donax* phytochelatin synthase 3; anaPCS = Anabaena PCC 7120 phytochelatin synthase; AsPCS1 = arsenate-coupled phytochelatin synthase 1; AtMRP3 = *Arabidopsis* Multidrug Resistance-associated Protein 3; AtPCS1 = *Arabidopsis* phytochelatin synthase 1; AtPCS2 = *Arabidopsis* phytochelatin synthase 2; BnPCS1 = *Boehmeria nivea* phytochelatin synthase 1; BSO = buthionine sulfoximine (PC biosynthesis inhibitor); cad1 = gene encoding AtPCS1 (*Arabidopsis* phytochelatin synthase 1); cad1-1 to cad1-6 = allelic series of cad1 mutants; cad1-3 = mutant allele of cad1 with impaired PC synthesis and enhanced Cd sensitivity; mutant containing a defective *AtPCS1* gene; CAD1 = gene encoding AtPCS1; Cd = cadmium; CdPCS1 = *Cynodon dactylon* phytochelatin synthase 1; CePCS = *Caenorhabditis elegans* phytochelatin synthase; Col-0 = Columbia-0 ecotype of Arabidopsis thaliana; EC = synthetic phytochelatin-like peptide (e.g., EC14, EC16, EC20); γ-EC = γ-glutamylcysteine; GSH = glutathione; GUS = β-glucuronidase; HMA2, HMA4 = Heavy Metal ATPases 2 and 4; IAR4 = IAA-Ala Resistant 4; IpPCS1 = *Ipomoea pes-caprae* phytochelatin synthase 1; LUC = luciferase; MnPCS1 = *Mimulus guttatus* phytochelatin synthase 1; MnPCS2 = *Mimulus guttatus* phytochelatin synthase 2; MpPCS = *Marchantia polymorpha* phytochelatin synthase; MT1a, MT2b = metallothioneins 1a and 2b; NPT = non-protein thiols; OsPCS1 = *Oryza sativa* phytochelatin synthase 1; OsPCS2 = *Oryza sativa* phytochelatin synthase 2; OsPCS5 = *Oryza sativa* phytochelatin synthase 5; OsPCS15 = *Oryza sativa* phytochelatin synthase 15; ox = overexpression line; PC = phytochelatin; PCS = phytochelatin synthase; PCs = phytochelatins; PP2A-4C = Protein Phosphatase 2A subunit 4C; PtPCS = phytochelatin synthase from an unspecified plant species; RNAi = RNA interference (gene silencing strategy); RT-PCR = reverse transcription polymerase chain reaction; SAIL_650_C12 = specific T-DNA insertion line for PCS1; SBP1 = Selenium Binding Protein 1; T-DNA = transfer DNA; TaPCS1 = *Triticum aestivum* (wheat) phytochelatin synthase 1; ThPCS1 = *Tamarix hispida* phytochelatin synthase 1; VIGS = virus-induced gene silencing; VsPCS1 = *Vicia sativa* phytochelatin synthase 1; YCF1 = Yeast Cadmium Factor 1; ZmPCS1 = *Zea mays* phytochelatin synthase 1.

## Supplementary Materials

The supporting information can be downloaded at: https://www.mdpi.com/article/10.3390/ijms26104767/s1.
