# Supplementary material for: Phytochelatins and Cadmium Mitigation: Harnessing Genetic Avenues for Plant Functional Manipulation
Source: Int J Mol Sci. 2025 May 16;26(10):4767. doi: 10.3390/ijms26104767 (PMC12112059; doi:10.3390/ijms26104767)
Supplement: Supplementary file 1 [file ijms-26-04767-s001.zip › ijms-3626321-supplementary.pdf]

Supplementary file 1

Phytochelatins and Cadmium Mitigation: Harnessing Genetic Avenues for Plant Functional Manipulation

Deyvid Novaes Marques [1]*, Cássio Carlette Thiengo [2], Ricardo Antunes Azevedo [1]

[1] Department of Genetics, Luiz de Queiroz College of Agriculture (ESALQ), University of São Paulo (USP), Piracicaba, São Paulo (SP), Brazil
[2] Luiz de Queiroz College of Agriculture (ESALQ), University of São Paulo (USP), Piracicaba, São Paulo (SP), Brazil
* Correspondence: deyvidnovaes@gmail.com

Search Script

To conduct a comprehensive bibliometric analysis of studies involving cadmium (Cd) and phytochelatins in plants, we utilized the Web of Science Core Collection database. We included only documents classified as "scientific articles," without any temporal restrictions (all years) (Figure 2). The search strategy employed Boolean operators and quotation marks to ensure the inclusion of relevant studies (n = 1395). To refine the results, we filtered the search to focus on plants and/or agricultural crops (n = 1155). Additionally, we excluded articles mentioning algae, as they were recurrent and did not fit the scope of our analysis (n = 1075). From this refined dataset, we identified two specific types of studies: those involving mutants (n = 93) and transgenics (n = 84). Since some studies overlapped, the search for mutants excluded articles related to transgenics and vice-versa, ensuring accuracy. Each set of articles was then individually evaluated, leading to further manual exclusions. After careful selection, the dataset for mutants comprised 39 articles of interest, while the dataset for transgenics included 44 articles.

The results from the advanced search in the Web of Science Core Collection were exported to Microsoft Excel® (.csv) and analyzed using VOSviewer 1.6.15 (Leiden, The Netherlands). This software was chosen for its capability to form clusters and connecting networks in bibliometric research. We used the bibliometric network to integrate data on authors, countries of publication, journals, institutional participation, and research trends. The integrated analysis of the scientific collaboration network and the set of information related to publication revealed a clear correlation between the number of publications and citation impact, highlighting distinct patterns of collaboration and scientific influence among countries.

1.1. Country participation and global collaborative networks (database mutants)

An analysis of global research participation by country revealed that 17 countries contributed original articles on the subject (Figure 3A). China emerged as the primary hub of knowledge production by volume, with 11 articles; however, its average citation rate (34.4) was relatively modest, surpassing only Poland (33.3) and Canada (25) among the top 10 producing countries (Table S1). In contrast, the USA and Australia, ranked second and third in publication volume, demonstrated substantially higher scientific impact, each with an average of over 200 citations per document. Germany matched Australia in publication count (6 articles) but had a lower average citation count (72.7), significantly trailing behind Italy (8th in production). Notably, European collaboration, especially among France, Belgium, and Poland, was prominent, while countries like Canada and the Netherlands exhibited less visibility and global impact. This underscores the importance of both publication volume and international collaborations in enhancing scientific impact.

When analyzed by clusters, Cluster 5 (USA and Australia) accounted for a significant portion of the field, representing 36% of total publications, underscoring their pivotal role in advancing and disseminating research on this topic. Cluster 3 (China, Germany, and Russia), which contributed 39% of all articles, displayed a balance in production and citations. Although China maintained strong connections with countries such as Australia and the USA, its average citation per article remained at 34.4. Cluster 2 (Spain, Belgium, and the Netherlands) demonstrated robust intra-European collaboration, representing 20.5% of the total publications. Cluster 4 (Poland, France, and Switzerland) contributed 18% of total publications, with Poland and Canada (also part of Cluster 4) standing out as central nodes in global collaboration. Conversely, Cluster 7 (Japan) had limited contributions and few visible collaborations, representing an outlier in terms of active international cooperation in this research domain.

1.2. Institutional Participation and Engagement (database = mutants)

A total of 71 institutions contributed to the 39 publications analyzed on this topic (Figure 3B). Collaborative patterns reveal clusters of closely aligned universities, while some institutions appear isolated. The University of Melbourne (Australia) and Purdue University (USA) lead in international collaboration, forming a strong bilateral partnership in both publication volume and citation impact (Table S2). In China, Nanjing Agricultural University collaborates with Zhejiang University, while Hefei University of Technology and South China Agricultural University operate more independently. In Europe, the partnership between the Leibniz Institute of Plant Biochemistry and the University of Bayreuth (Germany) is notable for its high impact, with 521

citations across six publications. Similarly, the University of Pisa (Italy) and the Swedish University of Agricultural Sciences (Sweden) maintain a significant collaborative relationship.

Some institutions, such as Hasselt University (Belgium) and the University of California, San Diego (USA), appear isolated in the network, lacking visible connections. This may suggest either a more independent research approach or collaborations not represented in this network analysis. In contrast, the Autonomous University of Madrid and the University of Castilla-La Mancha (Spain) are directly linked, each with two publications totaling 97 citations. Additionally, four Japanese universities—Tokyo University, Kyushu University, NARO Hokkaido, and NARO Tohoku—form an internally cohesive network, illustrating a notable intra-national collaboration despite Japan's absence of international ties within this field.

1.3. Most Influential Authors and Independent Collaborative Networks (database = mutants)

A total of 216 researchers contributed to studies on this topic, though only six had three or more publications (Table S3). Figure 3C displays collaborative networks, with clusters colored by collaboration or thematic overlap. The Dark Blue Cluster, featuring a dense network, includes key authors such as Andersen, C.R., Cobbett, C.S., Goldsbrough, P.B., and Howden, R. Among them, Cobbett, C.S. stands out with five publications and 1,560 citations, contributing to over 20% of the analyzed articles (39). The Orange Cluster is led by Clemens, S. (81.8 average citations across four articles). The Light Blue Cluster features prominent authors Vangronsveld, J., and Cuypers, A., who engage in reciprocal collaborations with Spanish authors in the Purple Cluster.

The Red, Yellow, and Green Clusters encompass more authors but yield fewer publications and citations. Notably, some authors, like Howden, R., have lower publication counts but significant citation averages, highlighting their impact. Authors such as Cobbett, C.S., and Goldsbrough, P.B., combine high productivity and influence, standing out as leaders in the field. Clusters include authors from multiple countries, emphasizing the role of international collaboration in advancing global research on mutants, cadmium, and phytochelatins, and underscoring the importance of networks for scientific progress.

1.4. Co-occurrence and Link Strength of Keywords by Authors (Database = mutants)

The bibliometric analysis of studies on cadmium and phytochelatins in mutants reveals key research trends and inter-topic connections (Figure 3D). "Phytochelatins" emerges as the most prominent keyword, with the highest frequency (20 occurrences) and total link strength (44), highlighting its central role and strong associations with "Glutathione" (32) and "Cadmium" (22)

(Table S4). "Cadmium" itself is notably frequent (11 occurrences), emphasizing concerns around its toxicity, while "Glutathione" appears 10 times, underscoring its relevance in antioxidant responses.

Model organisms and biological processes are also well-represented, with "Arabidopsis thaliana" appearing frequently (9 occurrences) and strongly linked (25) to terms such as "Cadmium Tolerance" and "Cadmium Stress" (6 and 5 occurrences, respectively). This reflects a focus on mechanisms of resistance and stress response. Temporal keyword analysis, visualized by publication year colors, reveals sustained interest in "Phytochelatins" and "Cadmium," while "Metal Homeostasis" and "Sulfate Transporter" suggest emerging research areas. Topics such as "Nutrient Homeostasis" and "Metal Homeostasis" are less frequent but essential, each occurring twice, while sulfur metabolism's importance is indicated by keywords like "Sulfate Transport" and "Sulfate Assimilation" (2 occurrences each). Additionally, "Reactive Oxygen Species" (2 occurrences) highlights the role of ROS in signaling responses to cadmium-induced oxidative stress.

1.5. Journals and Publishers in the Dissemination of Research (database = mutants)

Only nine journals published multiple articles on the topic throughout the recorded years (Table S5). *Plant Physiology* led with seven articles, amassing a total of 1222 citations (average 174.6 citations per article), underscoring its broad influence in the field. The *Journal of Experimental Botany* (113.3 citations per article), *New Phytologist* (156 citations per article), and *Plant, Cell and Environment* (47 citations per article) each hosted three publications. Among these nine journals, Oxford University Press and Wiley-Blackwell were the most active publishers, each with three journals contributing to the research. Elsevier and Springer also published on the topic, although each hosted only one journal. This diversity of publishers highlights widespread collaboration and dissemination efforts across various scientific publishing platforms.

2.1. Country participation and global collaborative networks (database = transgenics)

Eighteen countries were identified as contributors to the scientific literature on transgenics. China led with 15 articles (30% of total publications), followed by the USA with 9 articles (18%) (Table S6). Other leading countries had more modest contributions, ranging between 2 to 4 articles. Although China produced the highest number of publications, research from the USA and France exhibited much higher impact in citations, together accounting for over 60% of total citations (31.7% and 28.3%, respectively). France maintained the highest average

citation per publication (200), followed by the USA (138.2). India (121.5) and Spain (122) also showed significant citation averages, while Italy contributed notably with 79.3 citations per article.

Countries were organized into collaborative clusters (Figure 4A). The red cluster (France, Germany, Serbia, Poland) represented strong European collaboration, while the green cluster (USA, Italy, Spain, South Korea) illustrated transatlantic partnerships. France acted as a pivotal link across these major clusters. Smaller, distinct clusters emerged: the dark blue and yellow (China and Bangladesh), and purple (Japan and Thailand), demonstrated regional or bilateral cooperation that spanned continents, such as India and Australia in the dark blue cluster. The remaining countries exhibited individual or less detectable collaborative networks, reflecting diverse patterns of collaboration and scientific influence.

2.2. Institutional Participation and Engagement (database = transgenics)

Seventy-seven institutions were engaged in research publications on transgenics in the context of cadmium and phytochelatins, yet only 12 institutions produced at least two publications each (Figure 4B). Typically, institutions paired in collaboration formed notable research clusters, while others remained isolated. The University of California, Berkeley (USA) and INRA (France) led in terms of impact, achieving 5 publications and 853 citations, marking them as central to high-impact collaborative efforts (Table S7). In China, the Chinese Academy of Sciences and Shandong University also produced 5 publications, though their combined citation impact was comparatively lower (270 citations). Other Chinese institutions appeared to operate independently in their research efforts.

In Italy, collaboration between the University of Parma and the University of Rome La Sapienza was notable, with 5 publications yielding a substantial 487 citations, underscoring the quality of their collaborative output. Meanwhile, the University of Illinois (USA) operated without direct collaborations but stood out with 3 publications and 283 citations, reflecting significant individual impact.

2.3. Most Influential Authors and Independent Collaborative Networks (database = transgenics)

In analyzing the authors within this field, 230 researchers were identified, though only 10 had at least two publications with over 100 citations each (Table S8). Figure 4C illustrates collaborative networks among these authors, with colored clusters highlighting areas of thematic similarity and collaboration. Despite each listed author having two publications, citation impact varies widely. For example, the red cluster, containing the highest number of researchers (6),

forms a dense interaction network. In contrast, the yellow cluster (786 total citations) and blue cluster (464 citations) stand out with an impressive citation count due to collaborations between Jouanin, L. and Terry, N. (reflecting a strong US-France collaboration), and Korban, S. and Gasic, K. (US-Serbia), both central figures in their clusters. The green cluster shows a balanced influence with five prominent researchers from various nations, including Portugal, China, the USA, and India (Carreira L., Balish, R.S., Dhankher, O.P., Meagher, R.B., and Li, Y.), underscoring the notable global reach of US-based authors.

2.4. Co-occurrence and Link Strength of Keywords by Authors (database = transgenics)

The keyword "Cadmium" is most frequent (22 occurrences) and central in these studies, showing strong associations with "Phytochelatin" (39), "Phytochelatin synthase" (25), "Phytoremediation" (33), and "Hyperaccumulators" (11) (Figure 4D; Table S9). “Phytochelatin” appears 15 times, underscoring its role in metal detoxification, while "Glutathione" (10 occurrences) highlights its antioxidant importance. Among organisms, "Arabidopsis" (6 occurrences) is frequently studied, with links to “Cadmium Tolerance” and “Cadmium Stress” (6 and 5 occurrences). "Tobacco" also links to "Transgenic plants," suggesting its use as a model in cadmium response research. Clustering reveals connections between “Cadmium Tolerance” and “Cysteine synthase” (blue cluster), emphasizing cysteine biosynthesis in stress response. In the green cluster, “Thiols,” “electrochemical detection,” “metallothionein,” “brdicka reaction,” and “liquid chromatography” indicate a focus on analytical methods and compounds for metal detoxification.

2.5. Journals and Publishers in the Dissemination of Research (database = transgenics)

Nine journals published multiple articles on transgenics related to cadmium and phytochelatins (Table S10). Journal of Experimental Botany led with five articles and 292 citations (58.4 citations/article). Plant, Cell Tissue and Organ Culture followed with three publications and 100 citations (33.3 citations/article). Plant Physiology, with three articles totaling 799 citations (266.3 citations/article), demonstrated significant field influence. Noteworthy journals also included Journal of Agricultural and Food Chemistry (2 publications, 180 citations, 90 citations/article) and Plant Molecular Biology (2 publications, 204 citations, 102 citations/article). Planta had two articles amassing 228 citations (114 citations/article), while Journal of Plant Physiology and Gene showed averages of 59 and 26.5 citations per article,

respectively. Russian Journal of Plant Physiology had two publications but with only four citations, attributed to recent publications.

Among publishers, Springer stands out, covering four of the nine main journals (Plant, Cell Tissue and Organ Culture, Plant Molecular Biology, Planta, and Russian Journal of Plant Physiology), highlighting their role in advancing transgenic research with cadmium and phytochelatin studies through a high publication volume and substantial citations.

List of tables

Table S1. Top 10 countries with the most publications on *mutants* in studies involving *cadmium* and *phytochelatins*.

Countries	Total Publication	Total Citation	Average citation/publication
China	11	378	34.4
USA	8	1649	206.1
Australia	6	1558	259.7
Germany	6	436	72.7
Belgium	4	188	47.0
Poland	3	100	33.3
France	3	205	68.3
Italy	3	310	103.3
Canada	2	50	25.0
Netherlands	2	121	60.5

Table S2. List of the top 10 institutions publishing studies involving ‘cadmium’ and ‘phytochelatins’, focusing on ‘mutants’.

Name of Institution	Country	Total Publications	Total Citation
University of Melbourne	Australia	5	1557
Purdue University	EUA	4	1416
Nanjing Agricultural University	China	4	181
Leibniz Inst. Plant. Biochem.	Germany	3	265
Unoversity of Bayreuth	Germany	3	256
Hasselt University	Belgium	3	120
Hefei University Technol.	China	2	239
Univ. Calif. Sandiego	EUA	2	109
Univ. Autonoma Madrid	Spain	2	97
Univ. Castilla la Mancha	Spain	2	97

Table S3. Top authors with at least 3 articles with 'mutants' in studies involving 'cadmium' and 'phytochelatins' and their respective numbers of total citations and average citations per document.

Authors	Total Publication	Total Citations	Average Number of citations
Cobbett, C.S.	5	1560	312.0
Clemens, S.	4	327	81.8
Cuypers, A.	4	188	47.0
Goldsbrough, P.B.	4	1418	354.5
Vangronsveld, J.	3	120	40.0
Howden, R.	3	1211	403,7

Table S4. Keywords, frequency of occurrence and binding strength in studies involving 'cadmium' and 'phytochelatins' with a focus on 'mutants' (minimum number of occurrences of a keyword = 2).

Keywords	Frequency of occurrence	Total Link strength
Phytochelatin	20	44
Cadmium	11	22
Gluthathione	10	32
Arabdopsis thaliana	9	25
Cadmium tolerance	6	17
Cadmium stress	5	12
nutrient homeostase	2	7
Sulfate assimilation	2	7
Phytochelatin sintase	2	4
Reactive oxygen species	2	4
Sulfate transporter	2	4
Heavy metals	2	3
Metal homeostasis	2	2

Table S5. Journals with at least 2 publications in studies involving 'cadmium' and 'phytochelatins' with a focus on 'mutants'.

Journal	Total Publication	Total Citations	Average citation/Journal
Plant Physiology	7	1222	174.6
Journal of Experimental Botany	3	340	113.3
New Phytologist	3	468	156
Plant, Cell and Environment	3	141	47
Annals of Botany	2	97	48.5
BMC Plant Biology	2	116	58
Frontiers in Plant Science	2	45	22.5
Journal of Hazardous Materials	2	20	10
The Plant Journal	2	109	54.5

Table S6. Top 10 countries with the most publications on *transgenics* in studies involving *cadmium* and *phytochelatins*.

Countries	Total Publication	Total Citation	Average citation/publication
China	15	662	44.1
USA	9	1244	138.2
Italy	4	317	79.3
Germany	3	120	40.0
Japan	3	84	28.0
France	2	400	200.0
Spain	2	244	122
India	2	243	121.5
Poland	2	119	59.5
Czech republic	2	73	36.5

Table S7. List of institutions with at least 2 publications on *transgenics* in studies involving *cadmium* and *phytochelatins*.

Name of Institution	Country	Total Publications	Total Citation
University of California Berkeley	EUA	3	453
University of Illinois	EUA	3	283
University of Parma	Italy	3	250
Chinese Academy of Sciences	China	3	230
INRA	France	2	400
University Roma la Sapienza	Italy	2	237
University of Georgia	EUA	2	239
Zhejiang University	China	2	205
Beijing Forestry University	China	2	65
Southwest University	China	2	51
Shandong University	China	2	40
Russian Academy of Sicences	Russia	2	4

Table S8. Top authors (total more than 100 citations per 2 articles) on *transgenics* in studies involving cadmium and phytochelatins and their respective number of total citations and average citations per document.

Authors	Total Publication	Total Citations	Average Number of citations
Jouanin, L.	2	400	200.0
Terry, N.	2	386	193.0
Balish, R. S.	2	239	119.5
Carreira, L.	2	239	119.5
Dhankher, O. P.	2	239	119.5
Li, Y. J.	2	239	119.5
Meagher, R. B.	2	239	119.5
Gasic, K.	2	232	116,0
Korban, S.	2	232	116.0
Sanita di toppi. L.	2	115	57.5

Table S9. Keywords, frequency of occurrence and link strength on *transgenics* in studies involving *cadmium* and *phytochelatins* (Minimum numer of ocuurences of a keyword = 4).

Keywords	Frequency of occurrence	Total Link strength
Cadmium	22	50
Phytochelatin	15	39
Phytochelatin sintase	12	25
Phytoremediation	11	33
Tobacco	11	31
Gluthathione	10	28
Heavy metals	7	20
Arabidopsis	6	14
Cadmium tolerance	6	10
Transgenic plants	4	15
Thiols	4	12
Hyperaccumulators	4	11

Table S10. Journals with at least 2 publications on *transgenics* in studies involving *cadmium* and *phytochelatins*.

Journal	Total Publication	Total Citations	Average citation/Journal
Journal of Experimental Botany	5	292	58.4
Plant, Cell tissue and organ culture	3	100	33.3
Plant physiology	3	799	266.3
Gene	2	53	26.5
Journal of Agricultural and Food Chemistry	2	180	90
Journal of Plant Physiology	2	118	59
Plant Molecular Biology	2	204	102
Planta	2	228	114
Russian Journal of Plant Physiology	2	4	2
